# Clinical Characteristics, Treatments, and Outcomes of Patients with Myocardial Infarction with Non-Obstructive Coronary Arteries (MINOCA): Results from a Multicenter National Registry

**DOI:** 10.3390/jcm9092779

**Published:** 2020-08-27

**Authors:** Pawel Gasior, Aneta Desperak, Marek Gierlotka, Krzysztof Milewski, Krystian Wita, Zbigniew Kalarus, Joanna Fluder, Maciej Kazmierski, Paweł E. Buszman, Mariusz Gasior, Wojciech Wojakowski

**Affiliations:** 1 Department of Cardiology and Structural Heart Diseases, Medical University of Silesia, Ziolowa 47, 40-635 Katowice, Poland; joanna.fluder90@gmail.com (J.F.); kazmierski.maciej@gmail.com (M.K.); wojtek.wojakowski@gmail.com (W.W.); 2 3rd Department of Cardiology, School of Medicine with the Division of Dentistry in Zabrze, Medical University of Silesia, Katowice, Silesian Center for Heart Diseases, 41-800 Zabrze, Poland; acis777@gmail.com (A.D.); m.gasior@op.pl (M.G.); 3 Department of Cardiology, University Hospital, Institute of Medical Sciences, University of Opole, 45-401 Opole, Poland; marek.gierlotka@uni.opole.pl; 4 Centre for Cardiovascular Research and Development, American Heart of Poland, 43-450 Ustron, Poland; kpmilewski@gmail.com; 5 First Department of Cardiology, School of Medicine in Katowice, Medical University of Silesia, 40-635 Katowice, Poland; dl@gcm.pl; 6 Department of Cardiology, Congenital Heart Diseases and Electrotherapy, Medical University of Silesia, Silesian Center for Heart Diseases, 41-800 Zabrze, Poland; kalzbig@o2.pl; 7 Department of Epidemiology and Statistics, Medical University of Silesia, 40-055 Katowice, Poland; pbuszman@ka.onet.pl

**Keywords:** MINOCA, STEMI, NSTEMI

## Abstract

Background: Diagnosis of myocardial infarction with non-obstructive coronary arteries (MINOCA) requires both clinical evidence of acute myocardial infarction (AMI) and demonstration of non-obstructive coronary arteries using angiography. We compared the clinical features, treatments, and three-year outcomes in patients with MINOCA and myocardial infarction with obstructive coronary artery disease (MI-CAD). Methods: We retrospectively analyzed data for 205,606 hospitalized patients with AMI. MINOCA was indicated as a working diagnosis in 6063 patients (2.94% of all AMI patients). For the control group we included 160,886 patients with MI-CAD. We evaluated the baseline characteristics, medication management options, outcomes, and readmission causes at 36 months follow-up. Results: Patients in the MINOCA group were younger. Females constituted a greater proportion of patients in the MINOCA group when compared to MI-CAD patients. STEMI during admission was diagnosed less frequently in the MINOCA group when compared to the MI-CAD group. All-cause mortality at 12 months was higher in the MINOCA group (10.94% vs. 9.54%, *p* < 0.001). At 36 months, there was no difference in the all-cause mortality rates (MINOCA 16.18% vs. MI-CAD 14.93%, *p* = 0.081). All-cause readmission rates were lower in the MINOCA group when compared to the MI-CAD group at both 12 months (45.19% vs. 54.33%, *p* < 0.001) and 36 months follow-up (56.42% vs. 66.66%, *p* < 0.001). Conclusions: This is the first description of the clinical features, treatments, and three-year outcomes in a large population of Polish patients. The main finding of this study was a relatively low rate of MINOCA, with high rates of adverse events both at 12 and 36 months follow-up.

## 1. Introduction

Diagnosis of myocardial infarction with non-obstructive coronary arteries (MINOCA) requires both clinical evidence of acute myocardial infarction (AMI) and demonstration of non-obstructive coronary arteries using angiography (stenosis < 50%) [1,2].

Patients with MINOCA constitute 3% to 15% of all those with AMI [3,4,5]. The pathophysiology of MINOCA is multifactorial and poorly understood. Several different potential causes of MINOCA have been proposed, including those of microvascular (myocarditis, Takotsubo cardiomyopathy, coronary microvascular spasm, coronary microvascular embolism, and type 2 MI) or epicardial origin (coronary artery dissection, coronary artery spasm, or eccentric plaque). Additionally, patients with MINOCA appears to have less severe atherosclerosis (on angiography), tend to be younger, and are more frequently females, but are less likely to have hyperlipidemia as compared to the patients with myocardial infarction with obstructive coronary artery disease (MI-CAD). Furthermore, patients with MINOCA had favorable prognosis when compared to MI-CAD patients with less than 12 months mortality [5]. The discrepancy in prognosis between patients suffering from obstructive and non-obstructive MI may reflect differences in the underlying pathophysiological mechanisms, however equally may merely reflect differences in the risk factor profiles. This makes diagnostics and treatment of MINOCA challenging in daily clinical practice. Furthermore, recent data on outcomes of patients with MINOCA has been limited primarily to mortality. There is a scarcity of data regarding the health status and clinical profile of these patients.

Currently, there are no published data concerning long-term outcomes in a large population of MINOCA patients. This study aimed to compare the clinical features, treatment, and three-year outcomes in patients with MINOCA and MI-CAD.

## 2. Methods

### 2.1. Data Sources

A retrospective analysis of data was undertaken from three large registries: the Polish Registry of Acute Coronary Syndromes (PL-ACS), the Polish Nationwide Acute Myocardial Infarction Database (AMI-PL), and the Silesian Cardiovascular (SILCARD) registry. 

PL-ACS is a national, multicenter, prospective observational registry, which includes data on patients hospitalized with ACS in Poland [6]. In brief, PL-ACS is a joint project of the Silesian Center of Heart Diseases in Zabrze and the Polish Ministry of Health, in cooperation with the National Health Fund. The registry was founded in October 2003. In May 2004, the registry protocol was harmonized with the European Cardiology Audit and Registration Data Standards (CARDS). This analysis was undertaken in consecutive patients included in the registry in the calendar years 2006–2017. At that time, 414 hospitals were contributing to the registry. Data were collected by the treating physicians and entered into the electronic system of the registry. Data on post-hospitalization mortality, including the date of death, were obtained from the National Health Fund. 

The AMI-PL includes all cases of AMI that occurred between 2009 and 2014. The design for AMI-PL has been described earlier [7]. In brief, the database contains the record of all AMI cases provided by the National Health Fund, the sole public health insurer in Poland. The National Health Fund has signed contracts with private and public healthcare providers, and it is the only payer of medical procedures. Therefore, it provides unified electronic nationwide data on medical procedures and disease incidence. The AMI cases were selected based on a primary diagnosis coded in the International Classification of Diseases (ICD), Tenth Revision, as I21 or I22, irrespective of any AMI occurrence in the past.

The SILCARD database was based on the agreement between the Silesian Center for Heart Diseases in Zabrze and the Regional Department of National Health Fund in Katowice in order to conduct a comprehensive analysis of patients with cardiovascular diseases in the Silesian Province [8]. General information on the SILCARD database was previously reported. Briefly, the database contains records from all hospitals (*n* = 310) in the Silesian Province—a large administrative region in Southern Poland with a population of 4.57 million (roughly 12% of Poland’s total population), of which 3.80 million are adults [9]. The Silesian Province provides a well-developed hospital network with two tertiary cardiology hospitals, three cardiac surgery departments, and 20 catheterization laboratories. The National Health Fund provided all data for the database, covering the period between 2006 and 2016. The SILCARD database enrolled all consecutive Silesian adult patients hospitalized in the cardiology, cardiac surgery, vascular surgery, or diabetology units for any reason, or hospitalized in the internal medicine or intensive care units with the principal diagnosis of cardiovascular disease (CVD) [10]. CVD was defined as R52 or J96 or any I code according to the 10th revision of the ICD.

The institutional review board at each site approved all protocols. The approval of an ethics committee was not required for this study.

### 2.2. Study Population

The analysis included all patients from PL-ACS, AMI-PL, and SILCARD databases hospitalized with a principal diagnosis of ST-segment elevation myocardial infarction (STEMI) and non-ST-segment elevation infarction (NSTEMI) according to the current guidelines of the European Society of Cardiology. Data for all individual patients and all hospitalizations were analyzed. The outcomes during the 36 months were available for all included patients.

**Figure 1:**
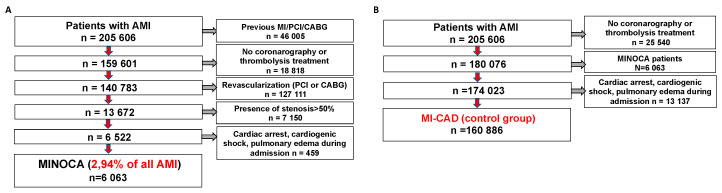
Study flowchart. (**A**) Minoca group flowchart (**B**) MI-CAD flowchart. AMI = acute myocardial infarction, MI = myocardial infarction, PCI = percutaneous coronary intervention, CABG = coronary artery bypass grafting, MINOCA = myocardial infarction with non-obstructive coronary arteries, MI-CAD = myocardial infarction with obstructive coronary artery disease.

Patients who were younger than 18 years at the time of hospitalization, had a history of acute myocardial infarction or percutaneous coronary intervention (PCI), or coronary bypass grafting (CABG) were initially excluded from the analysis. Only patients who underwent cardiac catheterization were included in our analysis. Additionally, patients who were assigned after coronarography to interventional treatment (PCI or CABG) or had > 50% stenosis in any epicardial artery were excluded. Furthermore, we excluded patients with either cardiac arrest, cardiogenic shock, or pulmonary edema during admission. The final cohort consisted of patients hospitalized for the first time due to AMI without prior history of any coronary revascularization with non-obstructive (< 50%) coronary stenosis (Figure 1A). For the MI-CAD control group, we excluded patients without coronarography or treated with thrombolysis prior to admission. Furthermore, we excluded patients with suspected MINOCA. as well as patients with either cardiac arrest, cardiogenic shock, or pulmonary edema during admission (Figure 1B). In our study, we compared the outcomes, numbers of hospitalizations, and distributions of cardiovascular disease entities up to 36 months in patients with diagnosed MINOCA and MI-CAD. The International Statistical Classification of Diseases and Related Health Problems (ICD) classification codes assigned to the individual disease entities are presented in Table 1.

### 2.3. Statistical Analysis

The long-term outcomes and repeated hospitalizations over a 36-month follow-up period were analyzed according to the first hospitalization of the given patient. Descriptive statistics were also applied. Qualitative variables were expressed as percentages. The comparative analysis was performed with the Chi square Pearson’s test. Continuous variables without normal distribution are expressed as the median with the interquartile range. The normal distribution of variables was verified by the Shapiro–Wilk test. The study groups were compared using the Mann–Whitney U test. The survival analysis was based on the Kaplan–Meier method. Statistica 13 software was used (Version 13.1, TIBCO Software Inc., Palo Alto, CA, USA). In order to adjust 12- and 36-month mortality to the differences in the baseline characteristics, the Cox proportional hazards model was used. Baseline characteristics factors that differed between the groups with *p* < 0.05 were analyzed by stepwise elimination (*p* < 0.05 to remain in the model). Results were presented as the hazard ratio (HR) with a 95% confidence interval (CI). Statistica version 13 (Version 13.1, TIBCO Software Inc., Palo Alto, CA, USA), a data analysis software system, was used for all calculations.

## 3. Results

Out of 205,606 hospitalized patients, 46,005 had a previous history of either AMI, PCI, or CABG; 18,818 patients did not undergo coronarography or received thrombolysis prior to admission; and a further 127,111 were treated with PCI or CABG. The remaining 7150 patients had > 50% stenosis in coronary arteries. Finally, we excluded 459 patients with cardiac arrest, cardiogenic shock or pulmonary edema during admission, leaving 6063 with a working diagnosis of MINOCA (2.94% of all AMI patients). For the control group, out of 205,606 hospitalized patients, 25,540 did not undergo coronarography or received thrombolysis prior to admission, while 6063 had a working diagnosis of MINOCA. Additionally, we excluded 13,137 patients with cardiac arrest, cardiogenic shock, or pulmonary edema during admission, leaving 160,886 patients with MI-CAD. 

Patients in the MINOCA group were younger (67 (58–77) years vs. 65 (57–75), *p* < 0.001). Females constituted 53.11% of the MINOCA group and 34.44% of the MI-CAD group (*p* < 0.001). STEMI during admission was diagnosed less frequently in the MINOCA group when compared to the MI-CAD group (16.55% vs. 49.09%, *p* < 0.001). Angina was the most common dominant symptom in both groups, however it was less pronounced in the MINOCA group (88.32% vs. 94.24%, *p* < 0.001). The median left ventricle ejection fraction was 50% (44.5–60) in the MINOCA group and 50% (40–55) in the MI-CAD group (*p* < 0.001). Only ~6% of patients were classified as NYHA class III or IV in both groups. Diabetes was less frequently diagnosed prior to the admission in the MINOCA group (22.46% vs. 25.86%, *p* < 0.001). The proportion of obese patients was lower in the MINOCA group (18.23% vs. 20.31%, *p* < 0.001). Sinus rhythm was present in 86.38% of MINOCA patients and 92.77% MI-CAD patients (*p* < 0.001). The baseline characteristics are summarized in Table 2.

Aspirin and P2Y12 inhibitors were prescribed less frequently in MINOCA when compared to MI-CAD patients (87.68% vs. 92.45%, *p* < 0.001; 67.58% vs. 84.80%, *p* < 0.001, respectively). Furthermore, patients from the MINOCA group received angiotensin-converting enzyme inhibitors and beta-blockers less often at discharge (74.01% vs. 79.39%, *p* < 0.001; 78.60% vs. 84.36, *p* < 0.001, respectively). In total, 83.38% of patients received statins in the MINOCA group when compared to 88.99% in the MI-CAD group (*p* < 0.001). Around 4% of patients received low molecular weight heparin injections in both groups. Oral anticoagulants at discharge were prescribed more frequently in the MINOCA group (5.89% vs. 2.18%, *p* < 0.001). The summary of medications at discharge is presented in Table 3.

**Figure 2:**
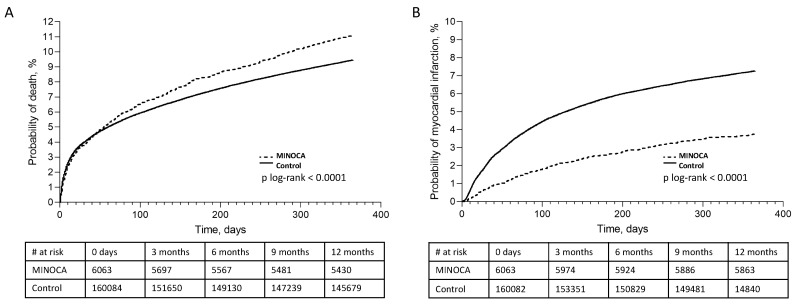
The 1-year Kaplan–Meier events rates. Kaplan–Meier curves show the cumulative incidence rates of (**A**) death and (**B**) myocardial infarction.

The in-hospital adverse events rates were generally low in both studied groups. Death occurred less frequently in the MINOCA group (1.67% vs 2.08%, *p* = 0.004). Additionally, cardiovascular death was lower in the MINOCA group (1.99% vs. 1.48%, *p* = 0.006). Cardiogenic shock and pulmonary edema developed less often in the MINOCA patients (respectively: 0.36% vs. 0.90%, *p* < 0.001; 0.25% vs. 0.58% *p* = 0.002). The recurrent MI was lower in the MINOCA group (0.06% vs. 0.38%, *p* < 0.001). There was no difference in the stroke rate during initial hospitalization. All-cause mortality at 12 months was higher in the MINOCA group (10.94% vs 9.54%, *p* < 0.001). At 36 months, there was no difference in the all-cause mortality (MINOCA 16.18%% vs. MI-CAD 14.93%, *p* = 0.081) (Figure 2A). Reinfarction rates were lower in the MINOCA group at both 12 months (3.83% vs. 7.26, *p* < 0.001) and 36 months follow-up (6.19% vs. 10.11, *p* < 0.001) (Figure 2B).

**Figure 3:**
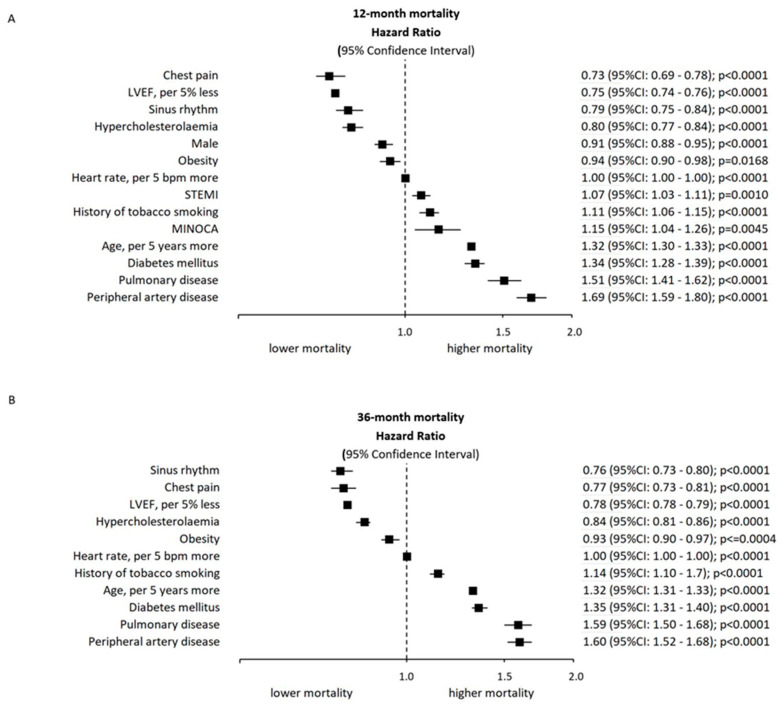
Multivariate analysis results. (**A**) 12 months results. (**B**) 36 months results.

Revascularization rates (either PCI or CABG) were significantly lower in the MINOCA group at both 12 months and 36 months follow-up. Cardiac ablation rates were higher in the MINOCA group at both time points (12 months: 0.96% vs. 0.18%, *p* < 0.001, 36 months: 1.32% vs. 0.29%, *p* < 0.001). ICD and CRT-D implantation rates were significantly lower in the MINOCA group up to 26 months follow-up (12 months: 1.30% vs. 18.5%, *p* < 0.001; 36 months: 1.65% vs. 18.89%, *p* < 0.001). After correcting for the differences in the baseline characteristics between the MINOCA and MI-CAD groups, the multivariate analysis confirmed that MINOCA was associated with increased 12-month mortality (HR 1.04, 95%CI: 1.15–1.26). However, MINOCA was not an independent factor associated with increased 36-month mortality (HR 1.02, 95%CI, 0.94–1.11). The results of the multivariate analysis are summarized in Figure 3. Patient outcomes are presented in Table 4.

All-cause readmission rates were lower in the MINOCA when compared to the MI-CAD group at both 12 months (45.19% vs. 54.33%, *p* < 0.001) and 36 months follow-up (56.42% vs. 66.66%, *p* < 0.001). Cardiovascular readmissions were less frequent in the MINOCA group up to 36 months (39.19% vs. 52.13%, *p* < 0.001). Chronic coronary syndrome was the most common cause for cardiovascular (CV) readmission in MINOCA and MI-CAD patients at both 12 and 36 months. Heart failure and cardiomyopathy rates were more frequent in the MINOCA group at both timepoints (12 months: 17.93% vs. 9.80%, *p* < 0.001; 36 months: 17.59% vs. 10.31%, *p* = <0.001, respectively). Furthermore, rehospitalization rates due to arrhythmia were significantly higher in the MINOCA group (12 months: 14.01% vs. 9.80%, *p* < 0.001; 36 months: 13.92% vs. 4.33%, *p* < 0.001, respectively). All readmission causes are summarized in Table 5.

## 4. Discussion

The present study describes the clinical features, treatments, and three-year outcomes in Polish patients with MINOCA. The main finding of this study was a relatively low rate of clinical diagnosis of MINOCA in MI patients, with high rates of adverse events and readmissions at both 12 and 36 months follow-up. 

As previously stressed by experts, MINOCA diagnosis should be treated only as a working diagnosis, which requires further examinations to clarify the underlying cause of the clinical presentation [11]. The exact MINOCA prevalence rate in the MI population differs among various studies and has been reported to be present in approximately 3–15% of cases [3,4]. In our study, the prevalence of MINOCA was 2.94%, which is in the lower end of the spectrum compared with previously published results. Furthermore, in accordance with the previous results, STEMI was approximately 3 times less frequent in patients with MINOCA (~17%) when compared to MI-CAD patients (~49) [5]. In our study, high rate of MINOCA patients suffered from hypertension (74%), were smokers (47%), and had diabetes (22%). Therefore, due to large proportions of patients suffering from risk factors, thrombosis and atherosclerosis caused by traditional risk factors cannot be excluded. Additionally, the atherosclerotic burden might have been missed due to low rates of intravascular imaging procedures during the analyzed period. However, the importance of intravascular imaging in the AMI setting was not stressed until the 2017 European Society of Cardiology guidelines for the management of STEMI, and we only analyzed patients hospitalized up to 2016 [12]. Furthermore, previous intravascular imaging studies have demonstrated evidence of atherosclerotic disruption in 40% of patients with MINOCA [13,14]. On the other hand, the left ventricular ejection fraction was not reduced, which might indicate a low degree of myocardial damage.

Conventional strategies for secondary MI prevention might not be suitable for all MINOCA patients due to various potential pathological mechanisms underlying the condition. Previous studies have found that MINOCA patients were less likely to receive secondary prevention therapy at discharge [15,16]. A previously published observational registry showed favorable outcomes after treatment with angiotensin-converting enzyme inhibitors, statins, and beta-blockers, while treatment with P2Y12 did not improve outcomes [17]. In our stud statins, angiotensin-converting enzyme inhibitors and beta-blockers were prescribed less frequently in the MINOCA patients when compared to MI CAD. Additionally, aspirin and P2Y12 inhibitors were prescribed less frequently in MINOCA when compared to MI-CAD patients. However, in the MINOCA group a relatively high proportion of patients received aspirin (87.68%), while only 65.58% of patients were prescribed with P2Y12 inhibitors. A large proportion of MINOCA patients are being treated as benign CAD because of a lack of significant obstructions in coronary arteries. This is most likely due to a lack of physicians’ knowledge of the appropriate management of MINOCA patients. In addition, the optimal pharmacological treatment has not been established yet in the guidelines, making it difficult to effectively treat MINOCA, resulting in confusion among physicians regarding the most beneficial secondary therapy for patients. It is important to emphasize that to date no data from randomized controlled trials are available to advise clinicians on best practices. This issue was also stressed in the Chinese population study, where a lack of appropriate guidelines left the physicians baffled regarding best therapy [18].

Due to the lack of obstructive atherosclerosis, it seems intuitive that the prognosis of MINOCA patients would be more favorable than in patients with MI-CAD. Furthermore, several studies have found a better prognosis of MINOCA when compared to MI-CAD patients [5,19,20,21,22]. On the other hand, only a few studies have demonstrated comparable outcomes for patients with MINOCA [23,24]. A contemporary meta-analysis reported a 12-month all-cause mortality rate of 4.7% in the MINOCA population, with a better prognosis than for those who experienced MI-CAD [5]. Data from a previously published large registry reported a 14% mortality rate during a mean follow-up of 4.5 years [25]. Our study demonstrated higher mortality at 12 months follow-up in the MINOCA group when compared to the MI-CAD group (10.94% vs 9.54%, *p* < 0.001). However, at 36 months follow-up there was no statistically significant difference in the mortality between MINOCA and MI-CAD groups (16.18%% vs. MI-CAD 14.93%, *p* = 0.081). Additionally, the multivariate analysis confirmed that MINOCA was associated with increased 12-month mortality. Nevertheless, MINOCA was not an independent factor associated with increased mortality at 36 months follow-up. Furthermore, in a large proportion of the studied MINOCA population, the initial diagnosis of increased cardiac biomarker levels due to cardiomyopathies might have been overlooked, which might partially explain the higher mortality when compared to the MI-CAD group at 12 months follow-up. Additionally, the overall higher mortality in the MINOCA patients in our study might be attributed to the older population of patients in our analysis (median 67 years) when compared to the mean age of MINOCA patients of 62 years in the previously mentioned registry. 

A previous study demonstrated that the rate of all-cause readmissions in patients with MINOCA was similar to the rate for those with MI-CAD (respectively 28.8% vs. 30%) [26]. The advantages of the presented study are 3 year follow-up and the exact evaluation of readmissions and their causes. In our analysis, all-cause readmission rates were approximately 10% lower in the MINOCA group when compared to the MI-CAD at both time points. Up to 36 months, the most common cause for cardiovascular (CV) readmission in both studied groups was a chronic coronary syndrome. However, this was approximately 20% less frequent in the MINOCA group when compared to the MI-CAD group. Heart failure and cardiomyopathies were significantly more frequent causes of cardiovascular readmissions at both 12 and 36 months follow-up in the MINOCA group (~18%) when compared to the MI-CAD patients (~10%). As mentioned before, the overlooked cardiomyopathies during the initial admission might explain the higher mortality in the MINOCA group at 12 months follow-up. Our results indicate that MINOCA is associated with a high rate of adverse outcomes. We stress the fact that it should be given the same attention as MI-CAD, although coronary arteries had showed obvious obstructions. 

### Study Limitations

First, in this study we used a generic definition of MINOCA, which included patients with suspected MINOCA. Furthermore, the recently published fourth universal definition of myocardial infarction [27] will change the context of acute myocardial infarction in terms of the MINOCA definition. Second, we do not have the data on how many patients underwent additional tests to determine the basic cause of MIINOCA. Third, our study is limited by its observational nature. Fourth, the absence of cardiac magnetic resonance, intracoronary imaging, pressure or doppler wire, and provocative spasm testing data may have impacted our results. Additionally, we do not have sufficient data for the test results in order to divide patients into subgroups based on the pathophysiological mechanism. Fifth, the core laboratory did not evaluate coronary angiographies, which were assessed at each hospital. Finally, the follow-up data were taken from the National Health Fund, so we did not have exact data on the causes of death in the studied population.

## 5. Conclusions

This is the first description of the clinical features, treatment, and three-year outcomes in a large population of Polish patients. A significant proportion of analyzed patients suffered from traditional CAD risk factors. Additionally, the majority of patients received conventional treatment for MI prevention. The major finding of this study was a relatively low clinical diagnosis of MINOCA in Polish MI patients, with high rates of adverse events and readmissions at both 12 and 36 months follow-up.

## Figures and Tables

**Table 1:** ICD codes for individual cardiovascular diseases.

**CAD**	I20, I21, I25
**HF and Cardiomyopathies (%)**	I42, I43, I50
**Arrythmias (%)**	I44, I45, I47, I48, I50
**Cerebrovascular Disease (%)**	I60, I61, I62, I63, I64, I65, I66, I67, I68, I69
**Hypertension (%)**	I10, I11, I12, I13, I14, I15
**Valvular Disease and Infective Endocarditis (%)**	I05, I06, I07, I08, I09, I35, I36, I37, I38, I39
**Disease of Arteries, Arterioles and Capillaries (%)**	I70, I71, I72, I73, I74, I75, I76, I77, I78, I79
**Other**	I26, I27, I28, I30, I31, I32, I40, I41, I51, I52, I80, I81, I82, I83, I84, I5, I86, I87, I88, I89, I90, I91, I92, I93, I94, I95, I96, I97, I98, I99

ICD = International Statistical Classification of Diseases and Related Health Problems, CAD = coronary artery disease; HF = heart failure.

**Table 2:** Baseline characteristics.

Variable	MINOCA *n* = 6063	MI-CAD *n* = 160,886	*p* Value
Age (years (Q1–Q3))	67 (58–77)	65 (57–75)	<0.001
Female (%)	53.11	34.44	<0.001
STEMI (%)	16.55	49.09	<0.001
**Dominant Symptom**			
Angina (%)	88.32	94.24	<0.001
Dyspnoea (%)	5.76	2.38	
Syncope (%)	1.40	0.53	<0.001
Fatigue (%)	1.09	0.69	<0.001
SBP (mmHg (Q1–Q3))	130 (110–150)	130 (110–150)	0.750
DBP (mmHg (Q1–Q3))	80 (75–100)	80 (75–100)	0.080
HR (1/min (Q1–Q3))	76 (70–90)	75 (68–85)	<0.001
LVEF (% (Q1–Q3))	50 (44.5–60)	50 (40–55)	<0.001
**Heart Failure**			
NYHA I (%)	62.56	62.54	0.730
NYHA II (%)	30.84	31.50	0.410
NYHA III (%)	4.43	3.89	0.042
NYHA IV (%)	1.81	2.07	0.210
Hypertension (%)	73.91	73.39	0.520
Hypercholesterolaemia (%)	35.85	44.19	<0.001
Smoking (%)	47.35	59.42	<0.001
Diabetes (%)	22.46	25.86	<0.001
Obestiy (%)	18.23	20.31	<0.001
Previous CAD (%)	7.27	13.93	<0.001
Previous PCI (%)	0	13.71	<0.001
Previous MI (%)	0	18.00	<0.001
Previous CABG (%)	0	3.91	<0.001
Previous stroke (%)	3.26	3.58	0.500
Previous kidney disease (%)	5.51	5.88	0.700
Previous lung disease (%)	4.42	3.68	0.009
Previous PAD	3.94	4.76	0.033
Sinus rhythm in ECG (%)	86.38	92.77	<0.001
Atrial fibrillation in ECG (%)	9.75	5.31	<0.001
Rhythm from pacemaker in ECG (%)	1.11	0.61	<0.001

MINOCA: myocardial infarction with non-obstructive coronary arteries; MI-CAD: myocardial infarction with obstructive coronary artery disease; STEMI = ST-segment elevation myocardial infarction; SBP = systolic blood pressure; DBP = diastolic blood pressure; HR = heart rate; LVEF = left ventricle ejection fraction; NYHA = New York Heart Association; CAD = coronary artery disease; PCI = percutaneous coronary intervention; MI = myocardial infarction; CABG = coronary artery bypass grafting; PAD = peripheral artery disease; ECG = electrocardiograph.

**Table 3:** Medications at discharge.

Variable	MINOCA *n* = 6063	MI-CAD *n* = 160,866	*p* Value
Aspirin (%)	87.68	92.45	<0.001
P2Y12 inhibitors (%)	67.58	84.80	<0.001
ACE inhibitors (%)	74.01	79.39	<0.001
Beta-adrenolytics (%)	78.60	84.36	<0.001
Statins (%)	83.38	88.99	<0.001
Nitrates (%)	9.09	12.44	<0.001
LMWH (%)	4.25	4.52	0.41
Oral anticoagulants (%)	5.89	2.18	<0.001

ACE = angiotensin converting enzyme; LWMH = low molecular weight heparin.

**Table 4:** Patient outcomes.

Variable	MINOCA *n* = 6063	MI-CAD *n* = 160,866	*p* Value
**In-Hospital**			
Cardiac arrest (%)	0.90	1.92	<0.001
Pulmonary edema (%)	0.25	0.58	0.002
Cardiogenic shock (%)	0.36	0.90	<0.001
Myocardial infarction (%)	0.06	0.38	0.004
Death (%)	1.67	2.08	0.004
Cardiovascular death (%)	1.99	1.48	0.006
Stroke	0.21	0.15	0.290
**12 Months**			
Myocardial infarction (%)	3.83	7.26	<0.001
Death (%)	10.94	9.54	<0.001
Stroke (%)	1.83	1.50	0.039
Coronarography (%)	6.93	26.02	<0.001
PCI (%)	4.37	20.33	<0.001
CABG (%)	3.10	5.48	<0.001
Cardiac ablation (%)	0.96	0.18	<0.001
ICD/CRT-D implantation (%)	1.30	18.15	<0.001
**36 Months**			
Myocardial infarction (%)	6.19	10.11	<0.001
Death (%)	16.18	14.93	0.081
Stroke (%)	3.10	2.64	0.030
Coronarography (%)	9.88	31.15	<0.001
PCI (%)	5.82	23.90	<0.001
CABG (%)	3.22	6.04	<0.001
Cardiac ablation (%)	1.32	0.29	<0.001
ICD /CRT-D implantation (%)	1.65	18.89	<0.001

PCI = percutaneous coronary intervention; CABG = coronary artery bypass grafting; ICD = implantable cardioverter–defibrillator; CRT-D = cardiac resynchronization therapy defibrillator.

**Table 5:** Readmissions at 12 and 36 months.

Variable	MINOCA *n* = 6063	MI-CAD *n* = 160,886	*p* Value
**12 Months**			
All cause readmission (%)	45.19	54.33	<0.001
Cardiovascular readmission (%)	31.30	43.62	<0.001
CAD (%)	45.27	73.23	<0.001
CCS (%)	25.91	45.63	<0.001
UA (%)	10.92	20.29	<0.001
STEMI (%)	3.97	3.78	0.840
NSTEMI (%)	4.47	3.53	0.110
HF and Cardiomyopathies (%)	17.93	9.80	<0.001
Arrythmias (%)	14.01	4.08	<0.001
Cerebrovascular disease (%)	5.34	2.40	<0.001
Hypertension (%)	5.24	2.26	<0.001
Valvular disease and infective endocarditis (%)	5.02	1.01	<0.001
Disease of arteries, arterioles and capillaries (%)	3.65	2.33	0.001
Difference	6.54	4.88	0.001
**36 Months**			
All cause readmission (%)	56.42	66.66	<0.001
Cardiovascular readmission (%)	39.19	52.13	<0.001
CAD (%)	44.66	69.59	<0.001
CCS (%)	24.46	41.99	<0.001
UA (%)	10.54	19.89	<0.001
STEMI (%)	4.52	4.42	0.890
NSTEMI (%)	5.14	3.80	0.026
HF and Cardiomyopathies (%)	17.59	10.31	<0.001
Arrythmias (%)	13.92	4.33	<0.001
Cerebrovascular disease (%)	5.94	3.25	<0.001
Hypertension (%)	6.19	2.71	<0.001
Valvular disease and infective endocarditis (%)	4.78	1.04	<0.001
Disease of arteries, arterioles and capillaries (%)	3.67	2.83	0.050
Difference	3.25	5.33	<0.001

CAD = coronary artery disease; CCS = chronic coronary syndrome; UA = unstable angina; STEMI = ST segment elevation myocardial infarction; NSTEMI = non-ST segment elevation myocardial infarction; HF = heart failure.
